# Current annotation strategies for T cell phenotyping of single-cell RNA-seq data

**DOI:** 10.3389/fimmu.2023.1306169

**Published:** 2023-12-21

**Authors:** Kerry A. Mullan, Nicky de Vrij, Sebastiaan Valkiers, Pieter Meysman

**Affiliations:** ^1^ Adrem Data Lab, Department of Computer Science, University of Antwerp, Antwerp, Belgium; ^2^ Antwerp Unit for Data Analysis and Computation in Immunology and Sequencing (AUDACIS) Consortium, University of Antwerp, Antwerp, Belgium; ^3^ Clinical Immunology Unit, Department of Clinical Sciences, Institute for Tropical Medicine, Antwerp, Belgium

**Keywords:** T cells, single cell, RNA-seq, annotation, bioinformatics, adaptive immunity, T-cell receptor

## Abstract

Single-cell RNA sequencing (scRNA-seq) has become a popular technique for interrogating the diversity and dynamic nature of cellular gene expression and has numerous advantages in immunology. For example, scRNA-seq, in contrast to bulk RNA sequencing, can discern cellular subtypes within a population, which is important for heterogenous populations such as T cells. Moreover, recent advancements in the technology allow the parallel capturing of the highly diverse T-cell receptor (TCR) sequence with the gene expression. However, the field of single-cell RNA sequencing data analysis is still hampered by a lack of gold-standard cell phenotype annotation. This problem is particularly evident in the case of T cells due to the heterogeneity in both their gene expression and their TCR. While current cell phenotype annotation tools can differentiate major cell populations from each other, labelling T-cell subtypes remains problematic. In this review, we identify the common automated strategy for annotating T cells and their subpopulations, and also describe what crucial information is still missing from these tools.

## Introduction

The first single-cell RNA sequencing (scRNA-seq) experiments started in 2009, and the technique became commercially available in 2014 (1). Single-cell RNA sequencing has rapidly gained widespread use, as more detailed information can be acquired using it than using bulk RNA-seq. Additionally, scRNA-seq data are becoming more accessible as more companies (e.g., 10x Genomics and BD Rhapsody^®^) are developing and optimising the technology, leading to a higher throughput and decreasing costs. With the increasing availability of scRNA-seq data, there has been a substantial increase in our understanding of the functions of immune cells. This has led to discoveries of new immune cell subpopulations, their dynamic and heterogeneous nature, and their role in disease (2–5). A particularly useful advantage of scRNA-seq for the study of the adaptive immune system is the ability to uncover paired information on the gene expression and the immune receptor of a single cell [more extensively reviewed in (6)]. However, defining the cellular profiles for adaptive immune cells remains a complex task. For example, the T cells of the adaptive immune system are very heterogeneous and can adopt a wide variety of phenotypes. In addition to a wide variety of phenotypes, there is an increased layer of complexity due to the highly polymorphic nature of the immune cell receptors they carry, such as the T-cell receptor (TCR) for T cells. The TCR is created through somatic recombination to create a highly variable CDR3 sequence containing a variable (V), and Junction (J) for alpha (α) and gamma (γ) chains, or a V, Diversity (D) and J for beta (β) and delta (δ) chains (7). These unique TCRs can recognise a vast array of epitopes, including immunopeptides, lipids, and some small molecules [e.g., phosphoantigens and Vitamin B metabolites (8)]. The most well-studied mechanism of epitope recognition is the antigenic peptide presentation by the major histocompatibility complex (MHC) protein, encoded in humans via the human leukocyte antigen (HLA) gene loci, and then to conventional αβ T cells (7). However, there are also unconventional T cells which are thought not to interact with MHC, such as mucosal-associated invariant T cells (MAIT), natural killer (NK) T cells, and γδ T cells (9). These unconventional T cells and their cellular profiles remain poorly understood.

A crucial step in the analysis of scRNA-seq data involves annotating the cells with the correct cellular phenotype. The initial manual annotation of the cells in a scRNA-seq dataset, after (pre-)processing, is time intensive, may contain data entry errors, and requires expert knowledge of the marker genes specific to the different cellular subsets. The initial (pre-)processing is commonly done using the R Seurat package (10) or the Python Scanpy package (11). For a more comprehensive description of the different steps in the (pre-) processing of scRNA-seq data, we refer you to this excellent review by Heumos et al. (2023) (12). In brief, the manual annotation of cells in scRNA-seq data is typically approached by clustering the cells and comparing these clusters to identify the differentially expressed genes (DEGs) among them to verify if they are known marker genes that are specific to cellular populations. This is hampered by a number of factors, however, such as a high gene dropout rate, the free-floating ambient mRNA of one cell being captured in a droplet together with another cell (droplet-based methods), or the poor expression of some marker genes at the RNA level, which would be better identified at the protein level (13). More recently, this manual annotation process has been superseded by automatic methods that leverage machine learning to automate and ease the burden (12). To aid in annotating cells with their phenotypes in scRNA-seq data, several automated pipelines have been developed to infer the phenotype based on a cell’s gene expression profile. However, these tools are often focused on inferring broader cell types (i.e., annotating a cell as a T cell), and it is unknown how well these tools work for inferring the subpopulations of these broader cell types (i.e., identifying a T helper [Th] 1 cell). Thus, in this review, we describe the currently available annotation tools for identifying T-cell phenotypes from scRNA-seq datasets. We compare their annotation strategies to the literature to verify whether they are fully capturing these hard-to-delineate subpopulations. Finally, we reflect on how well some of the unconventional T-cell populations are currently being captured.

## Single-cell annotation tools

To prevent the labour-exhaustive manual annotation of new datasets, automatic annotation tools have been developed to decrease time, improve labelling accuracy, and promote consistency. Automated annotation has become part of the current gold-standard approach to single-cell RNAseq, along with manual annotation/inspection of the automated annotations by expert review (i.e., expert familiarity with the common markers of cellular populations, which enables accurate annotation) (12). Therefore, a range of tools have been developed to aid in annotation automation (**Table 1**). As highlighted in **Table 1**, the current tools fall into several subcategories, each with distinct advantages and limitations. These annotation methods can also be distinguished by the type of machine learning (ML) approach, with methods categorized into unsupervised, supervised, or semi-supervised approaches.

**Table 1:** Common strategies and programs for annotating scRNA-seq datasets.

Method	Explanation	Labelling automation	Advantages	Limitations	Example
**Cluster based**	Manually annotating clusters of cells by expert based on the expression of certain marker genes	No	Transparent	Subjective Requires substantial prior knowledge Does not accord for sparsity of expression May miss sub-clustering patterns depending on initial chosen residuals	Seurat clusters (10)
**Marker gene-based**	Automated mapping of cell clusters based on the expression of a small set of marker genes	Yes	Transparent Requires little prior knowledge	Biased (batch effects) Quality of the annotation depends on “proximity” to the training data	Garnett (14)
**Gating- based**	Automated mapping of cell clusters based on the expression of a small set of marker genes	Yes/no	Transparent Can be tailorable to dataset Uses nearest neighbours to fill in sparsity of gene expression (i.e., kNN smoothing)	Requires substantial prior knowledge for new gating models	scGate (15)
**Gene set-based**	Classification based on a large set of gene expression markers. Typically trained on well annotated datasets of atlases	Yes	Harmonization of cell type definitions across studies Requires little prior knowledge	Not very transparent Biased (batch effects) Quality of the annotation depends on “proximity” to the training data	CellTypist (16), clustifyr (17)
**Reference-based/label transfer**	Map your data to existing reference and perform label transfer on the joint embedding	Yes	Allows you to (re)use cell type annotations from a previous dataset or experiment.	Impossible to take into account “new effects”. Requires strong degree of similarity between query and reference.	Azimuth (10), Symphony (18), scArches (19)

The unsupervised approach is typically clustering-based, including, for example, k-nearest neighbours (e.g., Seurat clustering (10)), which groups together cells with similar expression profiles. Subsequently labelling the clusters then requires the manual interrogation of the distinct markers per population. Accurate annotation relies on the expert knowledge of the user for common genes expressed for each cell type.

The supervised ML classification of scRNA-seq data is available in SingleR (20), Garnett (14), and CellTypist (16). These tools enable the prediction of cell-type labels for a novel dataset based on a prediction model trained on prior datasets. The ability to annotate a new dataset with high accuracy requires the dataset to have a good overlap of genes with the prediction model. This method is more robust in handling missing marker genes in a dataset, as it relies on the entirety of a cell’s gene expression to classify a cell, rather than just a few marker genes. However, if there is too much heterogeneity between the datasets, then the prediction tools fail to identify the cells correctly. The package scTriangulate aims to overcome this limitation by using multiple annotation sources (21).

The semi-supervised annotation approach includes models such as the SCINA (22) tool, which was developed to annotate cells based on a consensus list of known markers. An alternative tool, scGate (15), follows a process similar to the gating strategy employed in flow cytometry experiments, and classifies the markers in a hierarchical structure of pure and impure cells. The latter includes prelisted markers, adding to the interpretability of the method. The scGate researchers also defined common gating strategies on common cellular markers, and this led to the development of ProjectTILs (23) to further automate the process. A particular advantage of scGate is that the user can provide their own list of markers and and is advantageous to use in instances that the dataset is dissimilar or not modelled within the pre-learned supervised models.

Therefore, the researcher will need to consider which method is most appropriate for their dataset. For instance, if their dataset is similar to a previous annotated dataset and was obtained using the same technology, then the reference-based/label transfer may be the best strategy for annotating the cells. Alternatively, if researchers have a novel cellular subset from a species that is not human or mouse, the use of reference-based, gene set-based, and marker-based tools may not be advisable, as they rely on similarity to previously curated datasets. In addition, these ML-based label transfer methods are hampered by their reliance on the quality of the annotation of the original dataset. As such, we encourage users to carefully review the latest datasets and markers that were used to define populations, if available.

Although the accuracy of these automated methods has significantly improved, a two-step annotation process is strongly recommended. This two-step process involves primary annotations of the gene expression clusters by automated algorithms, followed by expert-based manual interrogation of the cell populations. In general, a combination of both strategies will result in the most accurate definitions of cell subsets.

## T-cell annotations

As highlighted above, the current annotation strategies can distinguish between populations with large phenotypic differences (e.g., B cell vs. T cells), as there are fewer overlapping transcripts. However, within each cell type there can be subspecialisations. For instance, T cells have a variety of subtypes. These subtypes are first stratified into two main lineages based on the TCR, that is, alpha-beta (αβ) and gamma-delta (γδ) T cells. Subsequently, αβ T cells, the best-described T-cell subtype, are further delineated into CD4^-^ or CD8^-^ expressing T cells. However, these can be further stratified based on their function and capacity for formation of immunological memory. The most well-described classical subpopulations relate to the class I (CD8^+^) and class II (CD4^+^) αβTCR cells, which are responsible for screening the peptide-loaded major histocompatibility complex for “self” and “foreign” antigens (7). Less is known about the unconventional T cells, which encompass natural killer T (NKT) cells, mucosal invariant T cells (MAIT), and γδ T cells. However, evidence that these unconventional T cells have important roles in both health and disease [reviewed in (24–26)] is emerging. Therefore, future work should consider both classical and unconventional T cells.

Given this plethora of cell subsets, how are these subpopulations currently defined by common annotation models for humans? To address this question, we looked at several tools that claim to be able to annotate for more delineated T-cell subpopulations. These annotation tools included scGate, CellTypist, and Data2Talk[online tool], as these had more extensive documentation for the T-cell subsets. Additionally, we compared these with the common protein expression panels used to identify T-cell subsets, as these are well-curated and validated panels. Last, we also included findings from the literature to fill in other annotation gaps. **Tables 2**–**5** highlight the markers used to classify the CD4 αβ T cells (**Table 2**), CD8 αβ T cells (**Table 3**), γδ T cells (**Table 4**), and miscellaneous T cell markers (**Table 5**) that were identified by the documented annotation models or through literature searches.

**Table 2:** CD4+ αβ T cell markers (human).

Type of T cell	Annotation tool	Feature set
**Th1**	CellTypist^	*CCL5, CXCR3*, and *TBX21*
	Data2Talk^&^	ND
	Flow panel^#^	TNFα, IFNγ, IL-2, CXCR3, and TBX21
	Flow panel^€^	CCR1, CCR5, CD3, CD4, CD8^-^, CD14^-^, CD19^-^, CXCR3, IFNγR1, IFNγR2, IL-12Rβ2, IL-18Rα, IL27Rα, STAT1, STAT4, T-bet, IFNγ, IL-2, TNFα, and TNFβ
**Th1/GZMK**	scGate	*GZMK, EOMES*, and *CRTAM*
**Th2**	Flow panel^#^	IL-4, IL-5, CCR4, and GATA3
	Data2Talk^&^	ND
	Flow panel^€^	CCR3, CCR4, CCR8, CD3, CD4, CD8^-^, CD14^-^, CD19^-^, CXCR4, IL-4Rα, IL17, RB, ST2/IL-33R, TSLPR, GATA-3, IRF4, STAT5, STAT6, IL-4, IL-5, IL-9, IL-10, IL-13, and IL-21
**Th9**	Flow panel^#^	IL-9, IL-10, and IRF4
	Flow panel^€^	CD3, CD4, CD8^-^, CD14^-^, CD19^-^, IL-4Rα, IL-17RB, TGF-β RII, IRF4, PU.1, CCL17, CCL22, and IL-9
**Th17**	scGate	*IL17A, IL17F, RORC, CTSH, KLRB1, CCL20*, and *IL26*
	CellTypist	*IL7R, CCR6*, and *ZBTB16*
	Data2Talk^&^	ND
	Flow panel^#^	CCR6, CD161 (*KLRB1*), IL-17, IRF4, and RORγt (*RORC*)
	Flow panel^€^	CCR4, CCR6, CD3, CD4, CD8^-^, CD14^-^, CD19^-^, IL-1RI, IL-6Rα, IL-21, IL-23, TGF-β RII, Batf, IRF4, RORα, RORγt/RORC2, STAT3, CCL20, IL-17A, IL-17F, IL-21, IL-22, and IL-26
**Tfh or Th21**	scGate	*IL21, CD200, CXCL13, TOX*, and *TOX2*
	CellTypist^	*PDCD1, ICOS*, and *CXCR5*
	Data2Talk^&^	ND
	Flow panel^#^	IL-21
	Flow panel^€^	BTLA, CD3, CD4, CD8^-^, CD14^-^, CD19^-^, CD40 Ligand, CD57, CD84, CXCR4, CXCR5, ICOS, IL-6 R α, IL-21 R, CD10, OX40, PD-1 (*PDCD1*), SLAM, CD150, Bcl-6, c-Maf, STAT3, CXCL13, IFNγ IL-4, IL-10, IL-17A, IL-17F, and IL-21
**Th22**	Flow panel^#^	IL-22, CCR10
	Flow panel^€^	CCR4, CCR6, CCR10, CD3, CD4, CD8^-^, CD14^-^, CD19^-^, CD161^-^, IL-6Rα, TGF-β RII, TNFRI, AHR, Batf, STAT3, CCL7/MCP-3, CCL15/MIP-1δ, FGFs, IL-10, IL-13, IL-21, IL-22, and TNF-α
**Regulatory T cells**	scGate	*FOXP3*
	CellTypist^	*CTLA4, IL2RA*, and *FOXP3*
	Flow panel^€^	CD73, CD3, CD4, CD5, CD14^-^, CD19^-^, IL-2Rα, ENTPD1, CD103, IL-7Rα low, CCLA-4, Folate Receptor 4, GITR, CD223, LAP, GARP, BDCA-4, CD134, CD62L, FOXP3, Helios(+/−), STAT5, Galectin-1, IL-10, IL-35, and TFG-β
**PD-1+ Tem/Effector Th**	CellTypist^	*PDCD1, CD4*, and *CTLA4*
**Tcm/Effector Th**	CellTypist^	*CD4, CCR7*, and *SELL*
**Memory CTL**	CellTypist^	*GZMK, CD4*, and *IL10*
**Tem/Effector Th**	CellTypist^	*KLRB1, AQP3*, and *ITGB1*
**Naive**	Literature (27)	CD25^−^ (*IL2RA*), CD45RA, CD45RO^−^, and CD127
**Teff**	Literature (27)	CD25, CD45RA(+/−), CD45RO(+/−), and CD127-
**Tem**	Literature (27)	CD25^−^, CD45RA^−^, CD45RO, and CD127
**Tcm**	Literature (27)	CD25, CD45RA^−^, CD45RO, and CD127
**MAIT**	Literature (28)	TRAV1-2 and CD161 (*KLRB1*), and IL-18Ra

Th, T helper; NK, Natural Killer; CTL, Cytotoxic T cells; Tfh, Follicular helper T cells; Tcm, Central memory; Tem, Effector memory.

^#^Flow cytometry protein expression panel markers from: https://www.biocompare.com/Editorial-Articles/569888-A-Guide-to-T-Cell-Markers/

^€^Marker summary from https://www.rndsystems.com/resources/cell-markers/immune-cells

^&^Bioturing can predict cell types based on 80,574,317 cells https://talk2data.bioturing.com/predict

^^^Curated markers from CellTypist (V2 list of markers).

ND, Not disclosed.

**Table 3:** CD8+ Markers (human).

Type of T cell	Annotation tool	Feature set
**CTL**	scGate	*HAVCR2, LAYN, LAG3, GZMB*, and *ENTPD1*
	Data2Talk^&^	ND
**Native**	Literature (27)	CD45RA, CD45RO^-^, CD62L, and CCR7
	Literature (29)*	*CCR7, SELL, IL7R*, and *TCF7*
	scGate	*LEF1, CCR7, TCF7, SELL, TOX-*, and *CXCL13-*
	Data2Talk^&^	ND
**Tcm/naive CTL**	CellTypist^	*CD8A, CCR7*, and *SELL*
**Tcm**	Data2Talk^&^	ND
	Flow Panel*^#^*	CCR7, CD127, CD62L, and IL2RA
	Literature (27)	CD45RA^−^, CD45RO, CD62L, and CCR7
**TEMRA**	scGate	*FCGR3A, CX3CR1*, and *FGFBP2*
	Data2Talk^&^	ND
**Tem/TEMRA CTL**	CellTypist	*CX3CR1, GZMB*, and *GNLY*
**Tem**	scGate	*GZMK* and *CXCR3*
	Data2Talk^&^	ND
	Literature (27)	CD45RA^−^, CD45RO, CD62L^−^, and CCR7^−^
**Teff**	Literature (27)	CD45RA, CD45RO^−^, CD62L^−^, and CCR7^−^
	Literature (29)*	*CD8A, GZMB, NKG7, GNLY*, and *GZMH*
**Tem/Teff**	Flow Panel^#^	HLA-DR, CCR5, TBX21, and GZMA
**Trm**	scGate	*ZNF683* and *ITGAE*
	CellTypist^	*ITGA1, ITGAE*, and *CXCR6*
**Tem/Trm CTL**	CellTypist^	*GZMK, CD8A*, and *CCL5*
**Tscm**	Literature (27)	CD45RA, CD45RO, CD62L, and CCR7
**Innate**	scGate	*FCER1G, IKZF2, TYROBP, KIR2DL3, KLRC3, KIR3DL2*, and *KLRC2*
**NKT**	CellTypist	*NKG7, GNLY*, and *CD8A*
	Literature	Vα24-Jα18 (TRAV10-TRAJ18) and Vβ11 (TRBV25)
**MAIT**	scGate	*TRAV1-2* and *SLC4A10*
	CellTypist^	*KLRB1, SLC4A10*, and *TRAV1-2*
	Literature (28)	TRAV1-2 and CD161, and IL-18Ra
**CD8αα T cells**	CellTypist^	*ZNF683, GNG4*, and *PDCD1*
**CD8α/β (entry)**	CellTypist^	*TOX2, SATB1*, and *CCR9*
**Precursor-exhausted**	scGate	*XCL1, XCL2, TOX, GNG4*, and *CD200*

CTL, Cytotoxic T cell; Tcm, central memory; Tem, effector memory; Teff, effector; Tscm, memory stem T cell; MAIT, Mucosal invariant T cells; NK, Natural Killer; Trm, Tissue resident memory; TEMRA, Terminally differentiated effector memory T cells.

^#^Flow cytometry protein expression panel markers from: https://www.biocompare.com/Editorial-Articles/569888-A-Guide-to-T-Cell-Markers/

^&^Bioturing can predict cell types based on 80,574,317 cells https://talk2data.bioturing.com/predict

^^^Curated markers from CellTypist (V2 list of markers).

**Figure 1:**
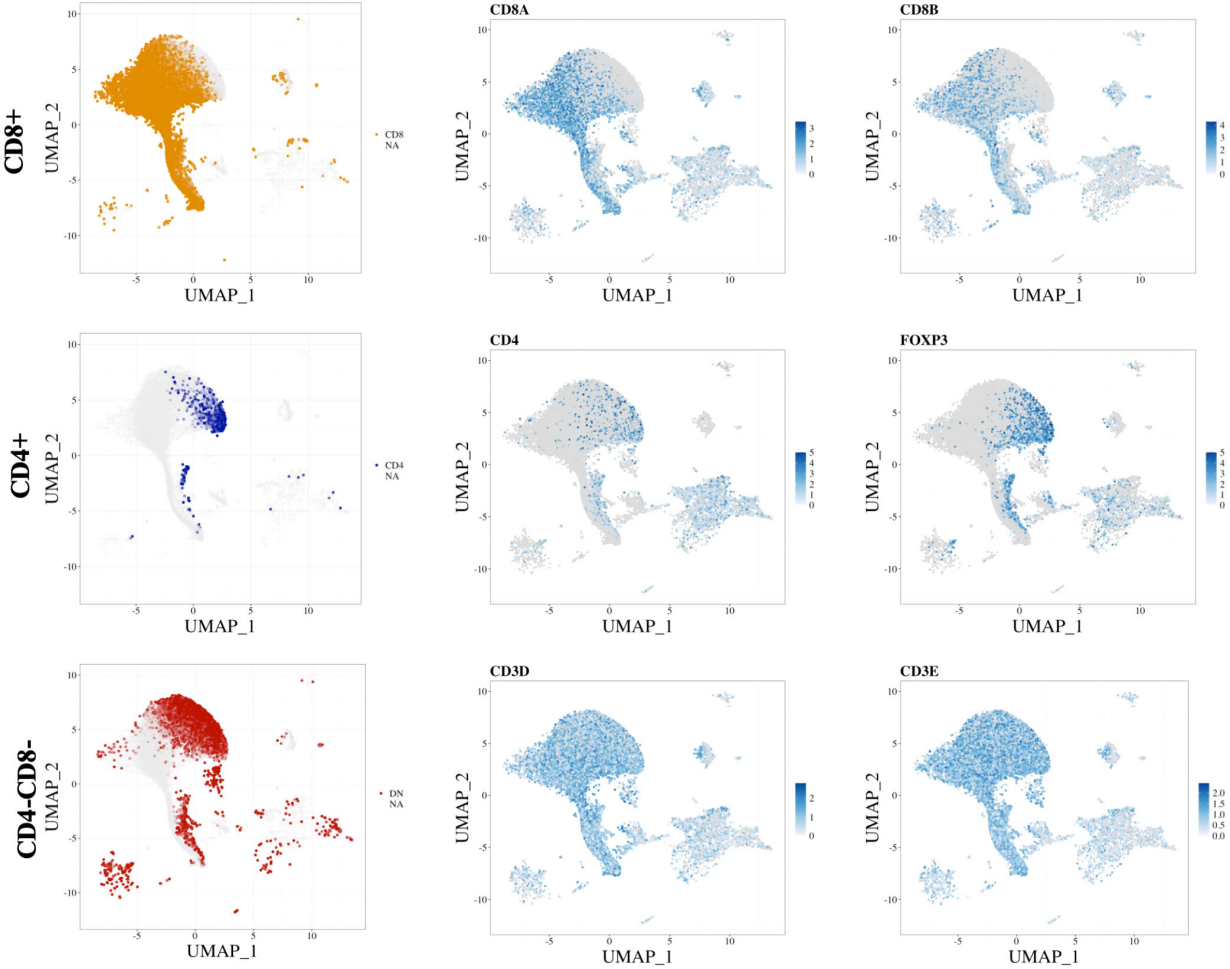
Marker sparsity of common T cell markers. represent the (top row) CD8^+^ T cells, (middle row) CD4^+^ T cells and (bottom row) double negative. The (left column) represents the scGate annotations, while the (middle and right columns) show the scaled expression of the markers of individual transcripts with the name listed above. The data was derived from an esophageal cancer set: GSE145370 (47), with the data processed and figure made with the aid of STEGO.R (48).

*Based on **Figure 1** top associated markers from a single-cell study.

ND, Not disclosed.

**Table 4:** γδ T cell Markers (human).

Type of T cell	Annotation tool	Feature set
**γδ T cell**	scGate	*TRDC, TRGC1, TRGC2*, and *TRDV1*
	CellTypist^	*TRDC, TRGC1, CCL5*
	Literature (29)*	*TRDV1, TRGV3, TRDV2*
**Innate (CD8 panel)**	scGate	*TRDC, TRGC1, TRGC2, TRDV1, TRDV2*
**Activated Vδ1+**	Literature (30)	NKp44 (*NCR2*), NKp46 (*NCR1*), and NKp30 (*NCR3*)
**Activated**	Literature (31)	NKp30 (*NCR3*), CCL3, CCL4, and CCL5
**Vδ2γ9+**	Literature (30)	TRGV9, TRDV2, and NKG2D
**Activated Vδ2γ9+**	Literature (30)	TRGV9, TRDV2, NKG2D, TNFα (*TNF*), CD16, and CCL4/CCL5
**T17**	Literature (30, 32)	TRDC, TRGC1, IL-17, and IFNγ
**CTL**	Literature (30)	NKG2D, PFR1, GZMB, GNLY and possibly express: CD95L TRAIL, CD27
**Regulatory**	Literature (32)	FOXP3
**Naive**	Literature (30)	IFNγ in presence of IL-2/IL-15
	Data2Talk^&^	ND
**Tcm**	Data2Talk^&^	ND
**Tem**	Data2Talk^&^	ND
**Eff**	Data2Talk^&^	ND
**Exhausted**	Data2Talk^&^	ND
**MAIT**	Data2Talk^&^	ND
**Cycling γδ T cells**	CellTypist	*MKI67, TOP2A*, and *TRDC*
**CRTAM+ γδ T cells**	CellTypist	*ITGAD, TRDC*, and *IKZF2*

CTL, Cytotoxic T cells; Tcm, central memory; Tem, effector memory; CRTAM, Cytotoxic And Regulatory T Cell Molecule; Eff, effector; Trm, Tissue resident memory; MAIT, mucosal invariant T cells.

^&^Bioturing can predict cell types based on 80,574,317 cells https://talk2data.bioturing.com/predict

^Curated markers from CellTypist (V1 list of markers).

*Based on **Figure 1** top associated markers from a single-cell study.

ND, Not disclosed.

**Table 5:** Miscellaneous T cell markers (human).

Type of T cell	Annotation tool	Feature set
**ETP**	CellTypist^	*ACY3, CD34*, and *SPINK2*
**DN thymocytes**	CellTypist^	*FXYD2, HES1*, and *CD99*
**Treg(diff)**	CellTypist^	*CD27, CCR7*, and *IKZF2*
**T(agonist)**	CellTypist^	*MIR155HG, BIRC3*, and *SMS*
**Early activation**	Flow panel^%^	CD69
**Later activation**	Flow panel^%^	CD25 (*IL2RA*)
**Very late activation**	Literature (33) and flow Panel^%^	HLA-DR^%^ (HLA-DRA, HLA-DRB5, and HLA-DRB1), CD38
**Senescence**	Literature (33)	CD57 (*B3GAT1*)
	Literature (34)	CD57 and KLRG1
**Exhaustion**	Literature (33)	PD1 (*PDCD1*)
	Literature (35)	TIGIT, CD279, LAG3, and PDCD1
	Literature (36)	Transcription factors panel of markers: TOX, NR4A, T-bet, EOMES, NFAT, IRF4, and BATF Inhibitory receptors: PD-1 (*PDCD1*), LAG-3 and HAVCR2 (*TIM-3*)
	Literature (29)*	*HAVCR2, PDCD1, LAYN, TOX, ITGAE, CTLA4, LAG3, ENTPD1, TIGIT*, and *CXCL13*
**Cycling T cells**	CellTypist^	*MKI67, TOP2A*, and *CD3D*
**Proliferation**	Literature (35)	*MKI67* and *TYMS*
	Literature (29)*	*ASPM, TOP2A, UBE2C, MKI67, CDKN2A, CD70, CDK4*, and *CDK6*

DN, Double negative; ETP, Early thymic progenitors; Treg, regulatory T cell.

^Curated markers from CellTypist (V2 list of markers).

^%^Flow cytometry protein expression panel markers from: https://www.sartorius.com/en/applications/life-science-research/cell-analysis/flow-cytometry/immune-cell-function/t-cell-activation

^&^Bioturing can predict cell types based on 80,574,317 cells https://talk2data.bioturing.com/predict

^Curated markers from CellTypist (V2 list of markers).

*Based on **Figure 1** top associated markers from a single-cell study.

The T-cell annotation models include most of the well-defined effector CD4^+^ populations, including T helper 1, Th17, follicular Th (Tfh) and regulatory T cells (Tregs) (**Table 2**). CellTypist was the only annotation model to included memory markers for the CD4^+^ T-cell population, while Data2Talk included Th2 cells, but the markers were not disclosed. The CD8^+^ T cells were classified into cytotoxic T cells (CTL; granzymes [*GZMB*, *GZMK*, etc.], perforin [*PFR1*], granulysin [*GNLY*]), NKT cells (*KLR* gene family, *CD160*, etc.), and MAIT cells (**Table 3**). These CD8^+^ T-cell subsets were also broken down into memory features, naive, effector, effector memory (Tem), terminal memory (TEMRA), resident memory (Trm), and central memory (Tcm) (**Table 3**) cells. The three annotation models cover many of the common classical CD8^+^ and CD4^+^ T-cell populations, except for Th2, Th9, and Th22 cells. The identification of these populations has relied on cytokine expression. However, the current technology inadequately captures the transcription factors and cytokines (e.g., interleukins) (37). In addition, these populations may also be missed due to a bias in the experimental choices, that is, no focused Th2 specific single-cell experiments. Therefore, we need to identify appropriate markers for the transcriptional level before we can add them to the label transfer models.

T cells can also be defined by their functional states, which are not necessarily restricted to T-cell lineage (e.g., γδ TCR vs. αβ TCR), or a specific subtype (e.g., CD4, CD8, or DN). These functional features include activation (e.g., CD69 [early], CD25 [late] and CD38/HLA-DR [very late]), exhaustion (PD-1, TIGIT, LAG3, and TIM3) (36), senescent (CD57 and KLRG1) (34), and cell cycling/proliferation markers (**Table 5**). However, the current automated annotation includes only the cell cycling markers. Given that these functional features are important in determining if a T cell is functioning properly, they need to be included in annotation models to identify the most biologically relevant T cell clones. It should be mentioned that when a cell expresses a marker associated with activation, senescence, or exhaustion, it does not mean a cell is activated, senescent, or exhausted. For instance, exhaustion is a functional state characterized by multiple features, including not only the expression of a combination of inhibitory genes such as *PD-1*, *TIGIT*, *LAG3*, and *TIM3*, and others, but also a lack of effector capacity, that is, a lack of cytokine production or cytotoxic activity (38). Defining these states is even further complicated by the fact that certain genes are associated with multiple states. For example, sole *PD-1* expression can indicate an activated state, but it can also indicate a differentiation state to exhaustion, or be a marker of exhaustion if expressed together with other immune checkpoint genes (39). Similarly, when *KLRG1* is expressed together with *CD57*, this can point to T-cell senescence; however, *KLRG1* can also be a defining feature of antigen-experienced memory T cells when expressed by itself (40). Therefore, to accurately annotate the exhausted and senescent cellular states, identification of the expression (or lack thereof) of multiple markers is needed. Researchers need to carefully design their annotation panels and be transparent about what markers were used to identify the subpopulations.

Currently, γδ T cells have limited representation in the annotation models (**Table 4**). The γδ T-cell population is subdivided into innate (Vδ2γ9^+^) and adaptive-like (e.g., Vδ1^+^, Vδ2^+^Vγ9^-^, Vδ3^+^) γδ T cells. The most studied γδ T-cell subpopulation is that of the invariant innate Vδ2γ9^+^ T cells that respond to (E)-4-Hydroxy-3-methyl-but-2-enyl pyrophosphate (HMB-PP). However, the models fail to adequately differentiate between these innate and adaptive-like γδ T-cell subpopulations. For instance, the scGate general annotation model classifies γδ T cells into the innate T-cell population along with NKT cells. CellTypist classifies γδ T cells as γδ TCR or CRTAM^+^ γδ T cells. Only Talk2Data includes γδ T cell sub-populations, but the markers used for the classifications are unknown (**Table 4**). Therefore, at this point in time, fully capturing the diversity of γδ T-cell subsets in scRNAseq data analysis requires expert knowledge of marker genes.

From the literature we know that there are difficulties obtaining data from the adaptive γδ T cells. γδ T cells are challenging to study as no antigen-specific culturing methods (41) exist for them, they have a minority fraction in the blood (comprising up to 10% of all T cells) (40), and have a high prevalence in mucosal membranes (e.g., skin, liver, and intestines) (42). Nevertheless, we are slowly defining adaptive γδ T cells that have overlapping phenotypes with the αβ T cells. For instance, functional information derived from mouse models has been used to identify several phenotypes, including T17^+^ (IL-17 and Th17-like) and T1 (IFNγ and Th1-like) cells (43). Intriguingly, on average ~30% of γδ T cells express the CD8 marker (40). Importantly, recent studies show CD8^+^ γδ T cells exhibit peptide restriction, similar to classical αβ T cells (44, 45). Consequently, γδ T cells express the same cytotoxic T-cell markers as CD8^+^ αβ T cells (30). Therefore, CD8^+^ γδ T cells may be functionally indistinguishable from CD8+ αβ T cells. We also note that γδ T cells can interact with CD1 and MR1, but their molecular signature is not well defined (46). Overall, the innate γδ T cells can be readily identified by their TCR arrangement in single-cell experiments. To bridge the adaptive γδ T cells annotation gap, therefore, we need to use TCR information along with what is known about classical and unconventional αβ T cells.

In addition to the issues with γδ T-cell classification, there are also issues with annotating other unconventional populations, such as MAIT and NKT cells. MAIT cells exhibit MR1 restriction and the semi-invariant TCR arrangement of TRAV1-2 with *TRAJ33*, *TRAJ12*, or *TRAJ23*, often paired with either the *TRBV6* or *TRBV20* gene families. In flow cytometry experiments, TRAV1-2 and CD161 (*KLRB1*), IL-18Ra or CD26 are commonly used to identify the MAIT population; however, there can be individual variability (28). MAIT cells can also exhibit the expression of Th17 markers (RORγt and IL-17), in addition to Th1-like features (T-bet, IFNγ) (28). Moreover, the semi-invariant type I NKT cells are identified by an invariant pairing of Vα24Jα18 (*TRAV10-TRAJ18*) with Vβ11 (*TRBV25*) and exhibit CD1d restriction, while the type II NKT cells have highly variable TCR combinations, and little is known about what their lipid-restriction is (9). It appears that there may be many subtypes of NKT cells, including Th17-like, Th2-like (GATA3), and Th1 (T-bet) cells (9). Therefore, both MAIT cells and NKT cells cannot be distinguished from other T-cell subpopulations based on gene expression alone. The easiest way to identify MAIT cells and Type I NKT cells will be to utilise the scTCR-seq data layer in combination with the gene expression layer. However, the type II NKT cells cannot be accurately identified until we can identify if they have specific gene(s) that are distinct from the other T-cell subsets.

Other considerations for annotation at the single-cell transcriptome level concern the gene sparsity, low abundance of transcripts captured, and poor correlation of mRNA expression to protein expression for several markers. To illustrate these problems, we highlight a common issue with the identification of the CD4^+^ T-cell population due to the abundance and sparsity of CD4 cells being lower than those of CD8A and CD8B cells (**Figure 1**). For this, we used the publicly available dataset GSE145370 (47), which was derived from CD45^+^ sorted cells from oesophageal tumour and adjacent tissue. The 14 available samples (~108,000 single cells) were then processed through the STEGO.R pipeline (48). The low abundance and high sparsity of CD4 cells makes it difficult to distinguish the double negative (CD4^−^CD8^−^) T-cell population from the true CD4^+^ population (**Figure 1** **, left column**). As an illustration of missing populations, we looked at a common marker associated with CD4^+^ Tregs, *FOXP3*. The semi-automated method may miss many of the CD4^+^ T regulatory cells if CD4 is used in the annotation model. Therefore, other common CD4-specific markers, in the absence of CD8, may need to be used as a surrogate for correctly annotating CD4 subpopulations. Alternatively, the sorting of pure CD4^+^ T-cell subpopulations (i.e., Th1 and Th2), followed by bulk RNA-seq and differential expression analysis, may be required to identify new population specific transcriptional markers. This would aid in finding alternative transcriptional markers to identify CD4 subpopulations without the need to use the CD4 transcript for annotation purposes.

A combination of gene and protein expression layers could be used to resolve several of these annotation problems. This inclusion of a protein expression layer has been made possible by the cellular indexing of transcriptomes and epitopes through sequencing (CITE-seq), allowing the use of protein-specific antibodies within scRNA-seq. For instance, the issue of identifying CD4^+^ T cells that stems from low mRNA abundance could be resolved by the addition of CD4-specific antibodies to capture CD4 protein expression. Another such problem that could be resolved by CITE-seq is the identification of memory subsets within T-cell populations. CITE-seq resolves this by capturing the expression of two CD45 protein isoforms that originate from alternative splicing, CD45RO and CD45RA, to differentiate between naive T cells (CD45RA^+^/CD45RO^−^) and memory T cells (CD45RA^−^/CD45RO^+^) (10). However, while the inclusion of protein antibodies is easily able to resolve isoform expression, this task is not as simple as relying on RNA expression alone. The sequencing of isoforms typically requires full-length transcripts, and the read lengths required to cover these transcripts are not obtained by the commonly used short-read methods for scRNA-seq (49, 50). To illustrate this limitation, using CellTypist to annotate cells in a scRNA-seq experiment, we are currently unable to differentiate between a naive T cell and central memory T cell (**Table 3**). However, scRNA-seq with the CITE-seq has been able to identify the memory populations (51). Alternatively, long-read sequencing, for example by Oxford Nanopore Technologies (ONT) or PacBio, can be used for scRNA-seq, and can readily resolve splicing/isoform information. For instance, ONT-based single-cell RNA sequencing led to the detection of multiple CD45 isoforms, consistent with CITE-seq data (52). Thus, to properly annotate memory T-cell populations or other T-cell populations that are defined by protein markers with poor mRNA expression correlation, we will either need to include protein expression (e.g., CITE-seq), or sequence isoforms using techniques that capture the full length of a transcript, such as long-read sequencing.

An additional inconsistency between the protein expression and transcriptional profiling pertains to the degree of expression. With flow cytometry, protein expression can readily capture dose, including low, moderate, and high, based on arbitrary cut-offs. However, due to fewer transcripts being captured, there is limited capacity to have these grades of expression in scRNA-seq, and they can mostly only be differentiated by binary (e.g., present or absent) thresholds. For instance, CD127^low^ protein expression is a marker for Tregs; however, this would be an inappropriate transcriptional marker (**Table 2**). Therefore, when designing a panel of transcriptional phenotyping markers, the expert will need to consider this technological limitation.

Overall, the above analysis identified inconsistencies with marker choice (**Tables 2**–**5**), which represents a concerning issue regarding the reproducibility of these T-cell studies. Additionally, there was a plethora of missing annotations (e.g., for Th2 cells, γδ T cell phenotypes, and functional features). Consequently, if these missing annotations are essential to identifying the T cell associated with a particular disease(s)/pathology (e.g., infection, cancer, autoimmune disease, and transplantation), using the automated models will lead to the T-cell subset of interest being missed. Therefore, filling in the missing annotations will need to be done manually or by way of a semi-automated process using custom gene sets.

## Identifiable needs for future T-cell annotation strategies

T cells remain a challenging subset of immune cells to interrogate due to their complex and variable subspecialisations, together with the diversity of the TCR repertoire. There has been some progress made in the development of T cell-specific annotation strategies and in TCR repertoire interrogation [reviewed in (6)]. Technology has progressed to now include simultaneous scRNA-seq and scTCR-seq, which can capture both the *αβTCR* and *γδTCR* genes (e.g., 10x Genomics and BD Rhapsody). Both these layers of data are likely needed to identify the role individual T-cell clones are performing at a given time point. For example, scTCR-seq can capture the paired *αβTCR* or *γδTCR* sequence and identify if the clone was expanded. Clonal expansion may indicate whether or not a particular TCR has responded to an epitope/antigen. The functional state will also further indicate if it is worth undertaking further analysis of the T cell and enable bystander clones to be ruled out. This information is needed for functional validation so that sorting based on phenotype-specific biomarkers and TCR genes can be done, which in turn can eventually be used as immunotherapies (e.g., CAR-T or TCR-T) (53). Having access to both layers in the initial discovery single-cell experiment will decrease the time needed to identify the most biologically relevant T-cell clones.

A deep dive into the current annotation strategies identified that inconsistences exist in the subclassification of T cells, along with missing T-cell subsets. To rectify these phenotyping inconsistencies, we will need a central resource of well-curated classifications so we can estimate the robustness of the markers for any given T-cell subpopulation. We may need to consider not segregating the classification based on γδTCR vs. αβTCR, as new understanding is showcasing overlapping, if not identical, markers (**Tables 2**–**4**). To achieve this database, the T-cell community requires the development of a public repository for protein markers, bulk RNA-seq derived markers, and, if possible, scRNA-seq with scTCR-seq and protein antibody information. Once this is built, we can determine the most robust markers per T-cell subset. We believe this literature review provides a useful reference and may serve as a foundation in the realization of this effort.

Once a consistent gene-set list of markers is established, we need to tackle the remaining problems regarding how to efficiently interrogate scRNA with paired scTCR-seq data. To achieve this, expert T-cell functional knowledge and computational expertise will be needed. This could help in determining which T cells should be functionally tested, and may lead to groundbreaking discoveries that lead to novel T cell-based therapeutics or help guide patient management in current immunotherapy protocols.

## Conclusions

In this study, we presented a comprehensive review of the tools used to annotate T cells from scRNA-seq datasets and also analysed the single-cell derived TCR repertoire. There are a multitude of automated strategies used to annotate T cells. However, the biggest shortcomings are a lack of consistency among tools concerning the markers used to annotate the T cell subsets, leading to severe issues with reproducibility. To overcome this challenge, collation of the currently available T cell-based data should be stored in a single repository, and development of new tools that make use of this harmonised framework is needed. Without this progress, there will continue to be issues with reproducibility, which will hamper progress in the development of T cell-based therapies.

## Author contributions

KAM: Conceptualization, Data curation, Writing – original draft, Writing – review & editing. NdV: Data curation, Writing – review & editing. SV: Data curation, Writing – review & editing. PM: Conceptualization, Writing – review & editing.
